# A smart MMP-9-responsive hydrogel releasing quercetin for psoriasis therapy

**DOI:** 10.3389/fphar.2026.1810299

**Published:** 2026-09-28

**Authors:** Qinghua Lan, Leyi Zhang, Yu Wang, Haici Lan, Qianqian Hu, Xiayan Zhang, Songmei Luo, Yanyan Xu

**Affiliations:** 1 Department of Pharmacy, The Fifth Affiliated Hospital of Wenzhou Medical University, Lishui, China; 2 Department of Drug Preparation, Lishui Hospital of Traditional Chinese Medicine Affiliated to Zhejiang Chinese Medical University, Lishui, China; 3 College of Pharmacy, North Henan Medical University, Xinxiang, China; 4 College of Food and Health, Zhejiang Agricultural and Forestry University, Hangzhou, China

**Keywords:** hydrogel, MMP-9 responsive, psoriasis, quercetin, silk protein

## Abstract

**Introduction:**

Psoriasis is an autoimmune disease characterized primarily by chronic inflammation, with suboptimal clinical therapeutic outcomes. Quercetin, a bio-safe plant secondary metabolite, exerts anti‐inflammatory effects by modulating multiple signaling pathways and holds promise as a therapeutic candidate for psoriasis.

**Methods:**

Our preliminary studies demonstrated that silk protein (SF) derived from silkworm cocoons exhibits excellent biocompatibility and can form hydrogels, serving as an ideal drug carrier for skin applications. We loaded quercetin into the SF hydrogel system and systematically investigated the therapeutic efficacy of this composite formulation (QSF) against imiquimod-induced psoriasis models through in vitro and in vivo experiments.

**Results:**

The hydrogel exhibited desirable enzyme-responsive properties. When exposed to an in vitro microenvironment containing 10 nM MMP‐9, the hydrogel network rapidly disintegrated, achieving a cumulative quercetin release of 58.26% ± 7.64% within 24 h. The enriched quercetin in lesion tissues markedly reduced the positive expression levels of IL‐17, TNF‐α, and IL‐1β, with inhibition rates of 32.14%, 20.25%, and 33.71%, respectively, which effectively interrupted the inflammatory cascade and obviously alleviated skin desquamation and pruritus.

**Discussion:**

Collectively, the fabricated QSF hydrogel presents anti‐inflammatory and symptomatic relief effects, providing a feasible strategy and experimental basis for the development of novel topical preparations for psoriasis treatment.

## 1 Introduction

Psoriasis is a chronic inflammatory skin disease that is difficult to be cured completely, affecting more than 2% of the global population (Sugumaran et al., 2024). The clinical symptoms of psoriasis are diverse, mainly including erythema, dry and scaly skin, persistent itching and pain in the affected areas, etc., which bring a heavy burden to the physical and mental health of patients (Lwin et al., 2024). The specific pathogenesis of psoriasis has not been elucidated by current medical science and technology, but some studies have shown that the cytokine inflammatory circuit formed by the interaction between multiple immune cells and keratinocytes plays a key role in the occurrence and development of psoriasis (Shen et al., 2022). For instance, under abnormal stimulation, helper T cells 17 (Th17 cell) secrete large amounts of pro-inflammatory cytokines (such as IL-17,IL-21 and IL-22) that act on keratinocytes. When the concentration of cytokines exceeds the threshold, it can activate the adaptive immune response of the body. A large number of neutrophils are chemotactic to the skin tissue and complete the trans-endothelial migration (Petit et al., 2021). In this process, the matrix metalloproteinase 9 (MMP-9) secreted by neutrophils activates the ERK1/2 and p38 signaling pathways in vascular endothelial cells, mediates the degradation of vascular endothelial connections (such as VE-cadherin), and subsequently destroys their barrier integrity (Alves-Filho et al., 2021; Chen et al., 2021). The significant increase in vascular permeability leads to more immune cell infiltration, which further exacerbates the inflammatory response. Therefore, reducing the level of inflammatory factors in the lesion and blocking the vicious cycle of inflammation are the core therapeutic strategies to improve the pathological process of psoriasis.

Quercetin is a representative plant-derived metabolite. It can regulate signaling pathways such as MAPK, NF-κB and Nrf-2 to achieve anti-inflammatory effects and shows great potential in the treatment of chronic inflammatory skin diseases (Beken et al., 2020; Hasan et al., 2022; Wang and He, 2022). Although quercetin has a hydrophilic structure with five hydroxyl groups, it is still affected by the conjugated structure formed by its own three aromatic rings, showing relatively low water solubility (Alizadeh and Ebrahimzadeh, 2022). To address the poor solubility of quercetin, Zhang Y et al. developed a gel delivery system based on hydroxypropyl-β-cyclodextrin-modified liposomes (HPCD/Lip@gel) (Zhang et al., 2023). Experimental studies confirmed that HPCD/Lip@gel effectively enhanced the bioavailability of quercetin. Nevertheless, the dual encapsulation structure of the new dosage form slowed down the drug release rate, resulting in delayed onset and an inability to quickly relieve the symptoms of dry, itchy, and painful skin. In view of this, the development of novel quercetin formulations featuring simple manufacturing processes, significant therapeutic effects, and suitability for topical application was identified as a promising approach for the efficient clinical treatment of psoriasis.

Silk protein (SF) hydrogel is a kind of polymer material with excellent biosafety and broad application prospects. Its inherent skin adhesion performance and the characteristics of drug release provide an ideal platform for long-term retention and local delivery of drugs at skin lesion sites (Mao et al., 2017). It is worth noting that, thanks to its amphiphilic multi-block structure with a relatively high proportion of hydrophobic segments, silk protein hydrogels can provide strong binding sites and compatible environments for low-solubility phytochemicals, thereby achieving efficient loading of hydrophobic drugs (Aleemardani et al., 2023). In addition to its high drug-loading capacity, enzyme-responsive biodegradation and drug release is another advantageous property of SF. Brown J et al. found that matrix metalloproteinases (MMP_S_) can specifically recognize and cut specific amino acid sequences in silk protein molecules, thus triggering the enzymatic degradation process of silk material (Brown et al., 2015). Given the high expression of MMP-9 in the lesion areas of psoriasis (Noddeland et al., 2024), we have reason to believe that the traditional Chinese medicine hydrogel composed of SF and quercetin can achieve intelligent delivery and microenvironment-responsive release of quercetin, creating favorable conditions for the rapid accumulation of drugs at the lesion sites.

In this work, we prepared quercetin/silk protein (QSF) hydrogel with MMP-9 response by ultrasonic crosslinking for the treatment of psoriasis (Scheme 1). When the above quercetin topical preparations are applied to psoriasis lesion area, the hydrogel can rapidly relieve dryness and desquamation by virtue of its high-water content. Meanwhile, its biological design can respond to the MMP-9 level and achieve release of drugs through enzymatic hydrolysis of SF. With the accumulation of quercetin in the lesion site, the levels of inflammatory factors (IL-17, TNF-α and IL-1β) can be significantly reduced, thus effectively alleviating the local tissue inflammation and tissue damage.

**SCHEME 1:**
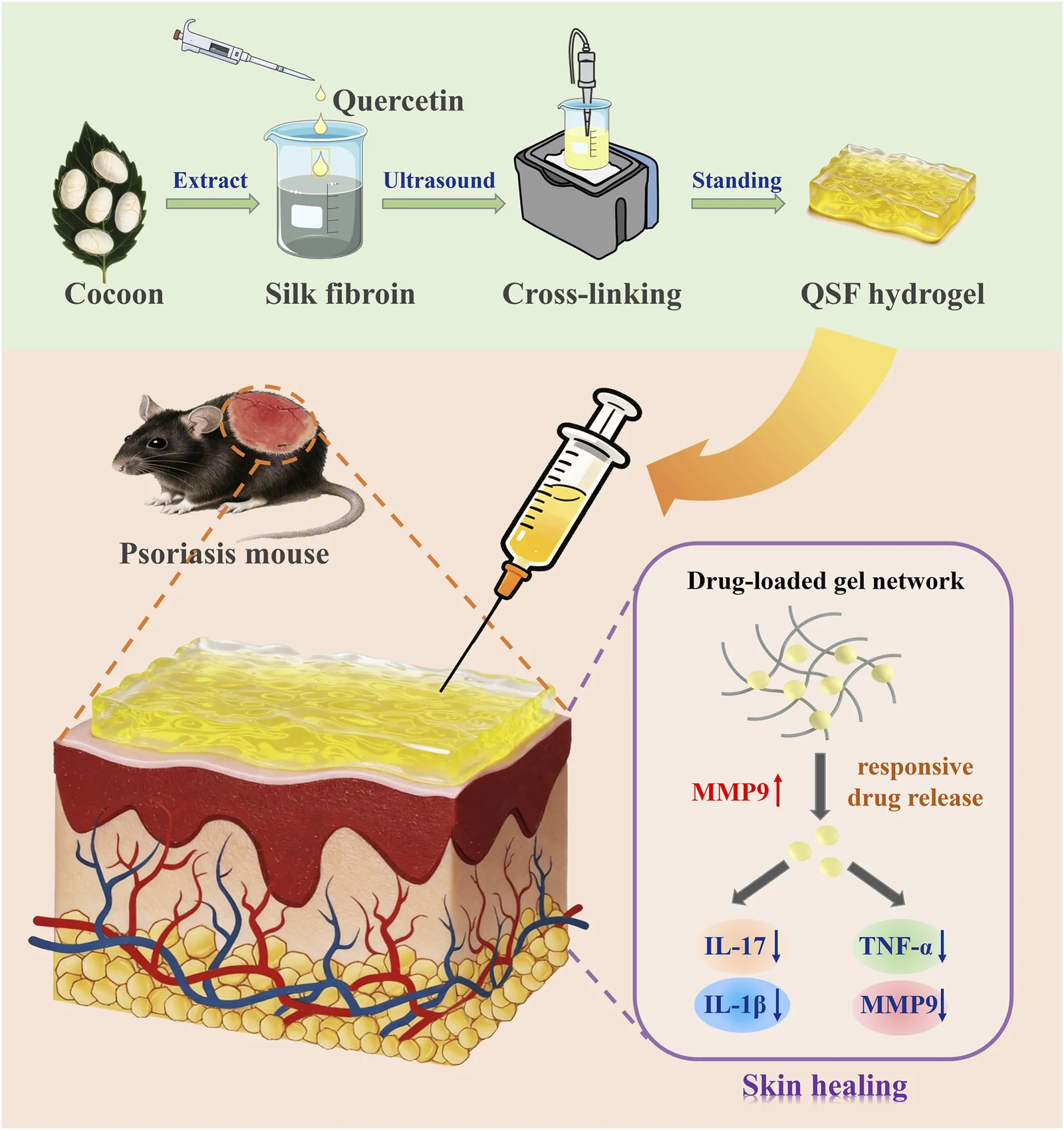
Therapeutic efficacy of MMP9-responsive quercetin/silk protein (QSF) hydrogel in a mouse model of psoriasis.

## 2 Materials and methods

### 2.1 Materials

The silkworm cocoons were supplied by the Xuzhou Hengtian Biotechnology Co., Ltd. (Jiangsu, China). Quercetin (Purity: HPLC ≥98%) was bought from Solarbio Science and Technology Co., Ltd. (Beijing, China). LiBr and Na_2_CO_3_ were provided by Maclean Biochemical Technology Co., Ltd. and Aladdin Biochemical Technology Co., Ltd. (Shanghai, China) respectively. Imiquimod Cream was obtained from Mingxin Pharmaceutical Co., Ltd. (Sichuan, China). Mouse IL-17A High Sensitivity ELISA Kit and Mouse TNF-α High Sensitivity ELISA Kit were acquired from Lianke Biotechnology Co., Ltd. (Hangzhou, China).

### 2.2 Preparation of QSF hydrogel

The extraction steps of SF include degumming, chain unfolding and purification (Yao et al., 2020). In short, the silkworm cocoons were cut into sheets and boiled in a Na_2_CO_3_ (0.02 M) solution for 30 min. Then, they were repeatedly washed with ultrapure water and dried in an oven. Next, the degummed SF was placed in a LiBr (9.3 M) solution and the chain was unchained in a water bath at 60 °C for 3 h. Then transfer the SF to the dialysis bag and replace the water in the dialysis tank at regular intervals. The purified SF solution was freeze-dried and stored for subsequent experiments.

SF was dissolved in ultrapure water at a concentration of 3% (wt./v), and an appropriate amount of quercetin solution was added to make the final drug concentration 50 μM. After vortexing the drug solution, the sample was labeled as 3%QSF. The 3%QSF was diluted with ultrapure water to solutions of 2%QSF and 1%QSF, and then placed on ice respectively. Samples were subjected to 100 W ultrasonic treatment for 4 min each to gel the drug solution.

### 2.3 Characterization of QSF hydrogel

The macroscopic and microscopic structures of QSF hydrogel were photographed respectively by camera and scanning electron microscope (SEM; Hitachi, SU8600, Japan). To explore the influence of physical crosslinking method on the functional groups of the two constituents of hydrogel, the samples were freeze-dried and mixed with an appropriate amount of KBr to form thin sheets, which were analyzed by Fourier transform infrared spectroscopy (FTIR; Thermofisher Scientific, Nicolet 5,700, USA). The ultraviolet-visible light spectrometer (UV-Vis; Pgeneral, TU-1901, Beijing) recorded the characteristic absorption spectra of quercetin, SF and QSF in the range of 200–450 nm.

The rheological properties of each group of hydrogels were tested using a rotational rheometer (DHR; TA, DHR-2, USA). Time-scanning measurements were conducted under fixed strain (γ = 0.1%), frequency (ω = 10 rad/s), and temperature (T = 37 °C). Frequency scanning measurements were conducted from 0.1 to 100 rad/s at a fixed strain (γ = 0.1%) and temperature (T = 37 °C). Within the shear rate range of 0.1 s^-1^ to 100 s^-1^, the variation law of the viscosity of the hydrogel with the shear rate was determined. Oscillation strain measurements were conducted at a fixed frequency (F = 1 Hz) and temperature (T = 37 °C), ranging from 0.1% to 320%.

### 2.4 *In vitro* drug release

Equal amounts of QSF hydrogel (0.1 g) were accurately placed into the upper chamber of Transwell inserts, while the corresponding lower chamber was filled with 1 mL of buffer solution containing MMP-9 at two concentrations (0 nM and 10 nM) as well as 2% (v/v) Tween 80. The entire Transwell release system was incubated in a constant-temperature shaking incubator at 37 °C with a continuous shaking speed of 120 rpm (Supplementary Figure S1). At predetermined time points, an aliquot of release medium was collected from the lower chamber, and an equal volume of pre-warmed fresh buffer containing the corresponding MMP-9 concentration was immediately replenished to maintain a constant volume and stable enzymatic activity of the release system.

### 2.5 Drug quantitative analysis

The *in vitro* release behavior of quercetin was quantitatively determined by triple quadrupole liquid chromatography-tandem mass spectrometry (LC-MS/MS; Waters Xevo TQD IVD, USA). High-performance liquid chromatography conditions: The chromatographic separation was performed on an ACQUITY UPLC BEH C18 column (2.1 mm × 100 mm, 1.7 μm). The mobile phase consisted of acetonitrile (phase A) and 0.1% formic acid aqueous solution (phase B), with a gradient elution program optimized as follows: 0 min, 10% A; 0.5 min, 40% A; 0.8 min, 90% A; 2 min, 95% A; 2.5 min, 10% A. The column temperature was maintained at 40 °C, the flow rate was set to 0.4 mL/min, and the injection volume was 5 μL. The mass spectrometric analysis was performed using an electrospray ionization (ESI) source in positive ion detection mode. The scanning range was set to m/z 100–1,500, with the following optimized parameters: Cone voltage of 60 V, Collison voltage of 34 V, ion source temperature of 150 °C, desolvation temperature of 500 °C, cone gas flow rate of 50 L/h, desolvation gas flow rate of 1000 L/h, and argon flow rate of 0.15 mL/min. Instrument operation and data acquisition were conducted using MassLynx V4.1 software.

### 2.6 Identification of DEGs

Gene Expression Omnibus (GEO) is a public database (http://www.ncbi.nlm.nih.gov/geo) (Edgar et al., 2002). We searched for relevant microarray datasets with the keyword “psoriasis”. The inclusion criteria were: The expression profiles were from the same sequencing platform GPL570 ([HG-U133_Plus_2] Affymetrix Human Genome U133 Plus 2.0 Array) and the test samples were derived from humans. Ultimately, the six datasets were obtained: GSE30999 (85 lesion samples and 85 adjacent normal tissues) (Suárez-Fariñas et al., 2012), GSE13355 (58 lesion samples and 58 adjacent normal tissues) (Nair et al., 2009), GSE14905 (28 lesion samples and 28 adjacent normal tissues) (Yao et al., 2008), GSE41662 (24 lesion samples and 24 adjacent normal tissues) (Bigler et al., 2013), GSE34248 (14 lesion samples and 14 adjacent normal tissues) (Bigler et al., 2013), and GSE50790 (4 lesion samples and 4 adjacent normal tissues) (Swindell et al., 2012). Using the Bioconductor package integrated in R software (version 4.4.0), the gene expression data between lesion samples and adjacent normal tissues were analyzed to identify significantly differentially expressed genes (DEGs). The criteria for determining the significance of DEGs were as follows: the corrected P-value was less than 0.05, and the absolute logarithmic fold change (|logFC|) was greater than 1.

### 2.7 Analysis of disease genes and drug target genes

The relevant targets of quercetin were obtained through the Traditional Chinese Medicine Systems Pharmacology Database and Analysis Platform (TCMSP), the online Drug Target Prediction Platform (PharmMapper), and Swiss Target Prediction (STP). The Venn diagrams were generated using R software to visualize the overlapping genes between DEGs associated with the disease (psoriasis) and the target genes of quercetin. Based on the intersecting genes identified in the Venn diagram, we established a protein interaction network (PPIs) using the STRING database. Finally, the Hub genes were screened by Cytoscape software and Gene Ontology and KEGG enrichment analyses were conducted by R software.

### 2.8 Cell culture

The reagents used in the cell culture experiment were provided by Thermo Fisher Scientific Co., Ltd. and the Human adult low Calcium High Temperature keratinocyte cell line (HaCaT) was purchased from the Cell Bank of the Chinese Academy of Sciences. HaCaT was cultured in complete medium (Dulbecco’s Modified Eagle Medium, fetal bovine serum, and penicillin/streptomycin at a volume ratio of 100:10:1), and the culture conditions were set in a constant temperature and humidity environment of 37 °C and 5% CO_2_.

### 2.9 CCK8 assay for cell viability

Since administration in gel state would affect the detection of cell viability, QSF solution was used instead of QSF gel to evaluate the biocompatibility of the new formulation. Briefly, the corresponding amounts of quercetin and silk protein were dissolved in complete culture medium, mixed thoroughly, and then sterilized by filtration through a 0.22 μm filter. HaCaT were inoculated into 96-well plates. After cell attachment, different drugs (SF, Q, QSF, IMQ, IMQ+Q, IMQ+QSF) were added and incubated for 24 h. The drug solution was then removed, followed by adding an appropriate amount of CCK8 solution and processing the cells for 2 h. Finally, absorbance values were measured at 450 nm wavelength using a multi-mode microplate reader (Tecan, Infinite M Plex, Switzerland), with calculations performed according to the formula specified in the manual.

### 2.10 Cellular immunofluorescence assay

HaCaT cells were cultured overnight in 6-well plates with coverslips, then treated with IMQ (100 μM), IMQ+Q (50 μM quercetin), or IMQ+QSF-containing media according to designated groups. After 24 h, the media was removed and cells were fixed with 4% polyformaldehyde. The cells were blocked with 5%BSA to reduce background, followed by incubation with specific primary antibodies at 4 °C overnight. Unbound antibodies were washed off with PBS, and the cells were incubated with fluorescently labeled secondary antibodies at room temperature in the dark for 1 h. Finally, DAPI was used for nuclear counterstaining, and images were observed and captured under a fluorescence microscope (Leica DMi8, Germany).

### 2.11 Quantitative real-time polymerase chain reaction (qRT-PCR)

Appropriate concentrations of HaCaT cells were seeded into 6-well plates, and the groups were set as Ctrl group, IMQ group (100 μM), IMQ+Q group (50 μM quercetin), and IMQ+QSF group. After administration by group, RNA samples were isolated using the Total cell RNA Extraction Kit (RE-03113, Foregene). The target samples were reverse transcribed into cDNA using the HyperScript III RT SuperMix for qPCR with gDNA Remover kit (R202-02, EnzyArtisan). Finally, cDNA, specific primers (Supplementary Table S1), and 2×S6 Universal SYBR qPCR Mix (Q204, EnzyArtisan) were pre-mixed and analyzed using a real-time fluorescent quantitative PCR system (Roche LightCycler 480 I, Switzerland).

### 2.12 *In vivo* anti-psoriasis study of QSF hydrogel

Male C57BL/6 mice (4–6 weeks) were obtained from the Experimental Animal Center of Lishui University (China Lishui). The mice were housed in a SPF-grade barrier system with a 12 h light/dark cycle, and the environmental temperature and humidity were set to 25 °C ± 2 °C and 55% ± 5%, respectively. This experiment adhered to animal welfare principles, providing the animals with a comfortable living environment, nutritionally adequate feed, and clean drinking water. The experimental protocol of this study was approved by the Animal Experiment Ethics Committee of Lishui University (No.2025D122).

After removing the fur from the dorsal skin of mice, they were randomly divided into four groups (n = 6), with the Ctrl group serving as a healthy control. The remaining three groups received imiquimod cream (62.5 mg per mouse) applied uniformly to the dorsal skin to induce psoriasis. For the treatment group, 12 h after imiquimod application, quercetin suspension (60 mg/kg) or QSF hydrogel was applied to the lesions using cotton swabs once daily for 1 week. Throughout the experimental period, daily PASI scoring was performed to evaluate disease severity, including skin erythema, thickness, scaling, and lesion extent. Each indicator was independently scored on a 0–4 scale: 0 indicates no clinical symptoms, 1 indicates mild, 2 indicates moderate, 3 indicates significant, and 4 indicates very significant. After the experiment, mice were euthanized and skin and spleen specimens were collected from the same areas. Skin samples were used for histological analysis, while spleens were weighed and subjected to pathological examination.

### 2.13 Histopathological examination

The standard protocols for H&E staining and immunohistochemistry were performed as described in the literature (Ning et al., 2025; Wang et al., 2025). Fresh skin and spleen tissues were fixed in 4% paraformaldehyde, then embedded in paraffin and sectioned. After dewaxing and hydration, the sections were stained with hematoxylin for nuclei and eosin for cytoplasm, followed by mounting. Immunohistochemistry procedures required antigen retrieval and non-specific site blocking on dehydrated sections. Subsequently, primary antibody, secondary antibody, and DAB chromogenic solution were applied sequentially, with final hematoxylin staining for nuclei before dehydration and mounting. Pathological slides were examined under a microscope and quantitatively analyzed using ImageJ software.

### 2.14 Detection of inflammatory factors by ELISA

Blood was collected from the ophthalmic sinus of mice and centrifuged in a 1.5 mL sterile tube at 3,500 rpm for 5 min. The supernatant serum was stored at-80 °C. The IL-17 and TNF-α levels in serum were quantified using a double-antibody sandwich assay, following the instructions of the corresponding ELISA kit.

### 2.15 Statistical analysis

All values are presented as mean ± standard deviation. The experimental data were statistically analyzed using GraphPad Prism 8.3 Software (GraphPad Software, San Diego, CA, USA). Statistical comparisons between groups were performed using ANOVA and Student's t-tests. A P value <0.05 was considered statistically significant (*P < 0.05, **P < 0.01, ***P < 0.001).

## 3 Results and discussion

Biologics, cyclosporine, methotrexate, and glucocorticoids are the representative medications in psoriasis treatment, which work by directly or indirectly suppressing inflammatory factors to alleviate skin lesions and control disease progression. However, psoriasis is a chronic condition with poor prognosis. Prolonged use of standard therapies may lead to heavy economic burdens, drug-induced liver and kidney damage, and secondary infections caused by extensive immunosuppression (Rapalli et al., 2020). Based on this, we incorporated the anti-inflammatory plant metabolite (i.e., quercetin) into the SF matrix to fabricate QSF hydrogel with a facile fabrication process and favorable biosafety.

### 3.1 Preparation and characterization of QSF hydrogel

From an external appearance, the premixed solution of quercetin and SF appears transparent and uniform yellow, with no obvious phase separation, indicating good compatibility between the two (Figure 1A). Studies have shown that the hydroxyl groups on the structure of quercetin can promote the gelation of fibroin, but this process requires a considerable amount of time (Aleemardani et al., 2023). To enable the rapid transition of QSF from a liquid to a gel state, this study employed ultrasonic physical cross-linking. As shown in Figure 1B, the QSF hydrogel exhibits poor fluidity, with its color changing as the concentration of quercetin increases, manifesting as a slight deepening of the milky yellow hue. Additionally, the alteration of the secondary structure (random curling to β-folding) is one of the important factors for the gelation of SF. Due to its heterogeneous microstructure, light scattering occurs in the visible light range, resulting in the transparency of the QSF hydrogel system being lower than that of the solution before gelation (Farokhi et al., 2021). Morphological analysis of SF and QSF hydrogels was performed using scanning electron microscopy. All hydrogels presented a porous structure, but the pores of 2%QSF hydrogels and 3%QSF hydrogels were relatively larger and smoother (Figure 1C). In response to this phenomenon, we speculate that the presence of quercetin may lead to an increase in pore size of the hydrogel. Larger pore structures can enhance the contact area between the carrier material and water, allowing QSF hydrogels to maintain higher moisture content, creating an ideal moist environment and water supply for rapidly alleviating the symptoms of dryness and desquamation in psoriasis (Chen et al., 2015; Zhang et al., 2025).

**FIGURE 1:**
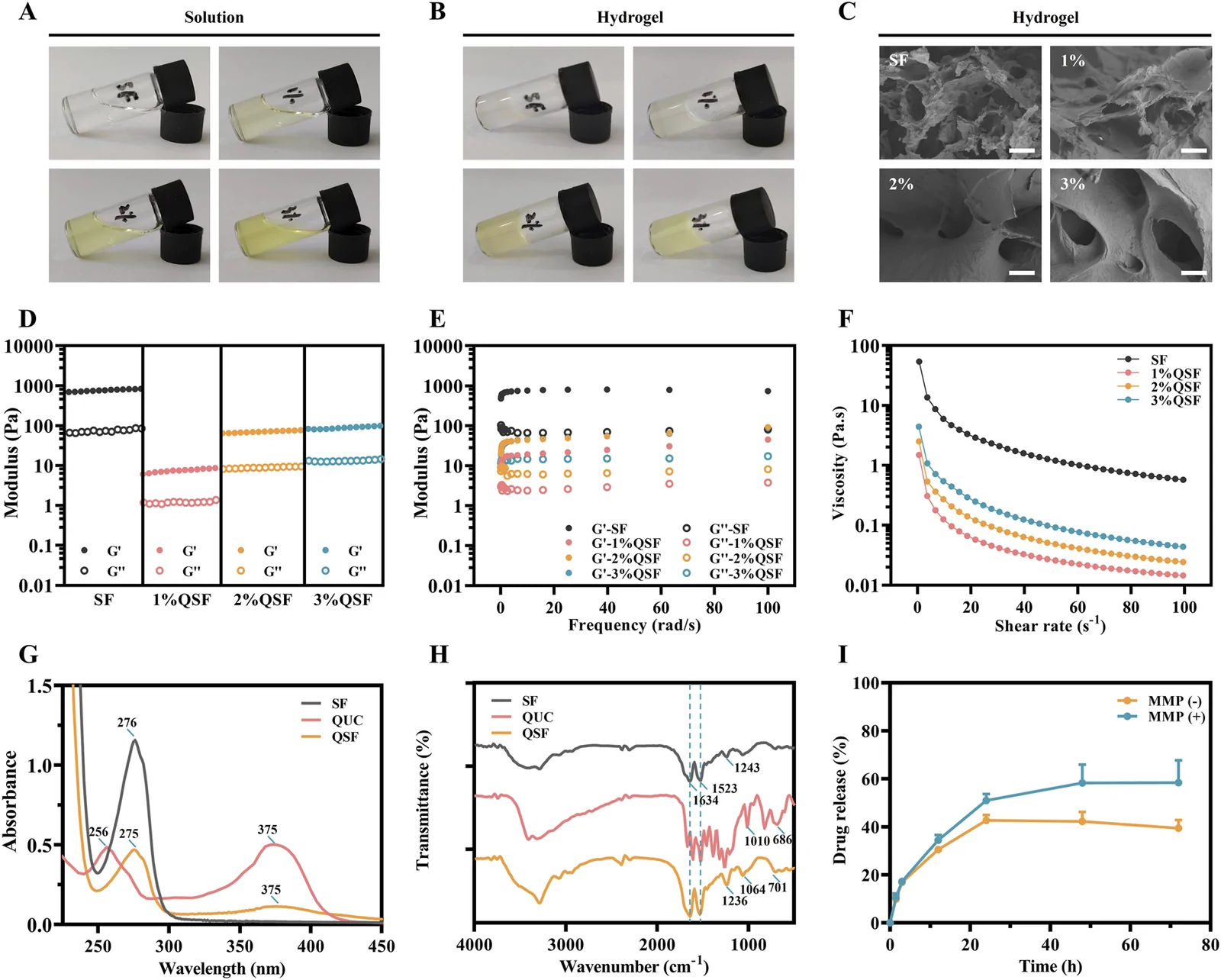
Morphology and rheology of QSF hydrogel. The photographs of the SF, 1%QSF, 2%QSF and 3%QSF in solution state **(A)** and gel state **(B)**. **(C)** SEM images of the SF, 1%QSF, 2%QSF and 3%QSF in gel state (scale bar = 10 μm). **(D)** The rheological behavior of QSF hydrogels swept by time. **(E)** The rheological behavior of QSF hydrogels swept by frequency. **(F)** The viscosity of QSF hydrogels with the shear rate from 0.1 to 100 s^-1^. The UV-Vis spectra **(G)** and FTIR spectrum **(H)** of the silk protein (SF), quercetin(Q), and QSF hydrogel. **(I)** The cumulative release of quercetin from the QSF hydrogels was measured after treatment for 72 h with or without 10 nM MMP-9. Data are presented as mean ± standard deviation (n = 3).

### 3.2 Rheological properties of QSF hydrogels

Based on the rheometer measurement data, curves were plotted to further investigate the rheological properties of QSF systems with different concentrations. The dynamic modulus variations of SF and QSF systems over time or frequency were shown in Figures 1D,E. Within the frequency range of 0–100 rad/s, the storage modulus (G ') of each hydrogel group was significantly higher than the loss modulus (G ″), indicating that the fibroin-based hydrogels exhibited primarily elastic behavior. This also suggests that the internal network structure of the QSF hydrogel is relatively stable, with strong intermolecular interactions (Zhang et al., 2024). Notably, compared to QSF hydrogels, SF hydrogels showed higher storage and loss modulus. This might be due to the loading of quercetin disrupting the fibroin network structure, leading to a decrease in the modulus of the drug hydrogel (Sun et al., 2024). Given that the G ' and G ″ of 2% and 3% QSF hydrogels were similar, this study chose the lower concentration of 2% QSF hydrogels as the subject for subsequent research. The viscosity curve graph (Figure 1F) indicates that all hydrogels exhibited shear-thinning rheological behavior, with higher concentrations of QSF leading to higher viscosity. In conclusion, 2% QSF hydrogel has excellent mechanical properties and injection performance, which is conducive to clinical application.

### 3.3 Spectral characterization of QSF hydrogel

Various spectroscopic images can analyze the structure, composition, and interactions of hydrogels at the molecular level. A deeper analysis of the 2% QSF hydrogel was conducted using UV-Vis spectroscopy and FTIR. As shown in Figure 1G, the free quercetin solution exhibited two distinct UV absorption peaks at 375nm and 256 nm. After adding SF, the drug characteristic absorption peaks in the system broaden. This phenomenon suggested possible intermolecular interactions between the carrier material and the drug (Putri et al., 2023). The IR spectrum of SF showed sharp peaks at 1,634 cm^-1^ (amide I), 1,523 cm^-1^ (amide II), and 1,243 cm^-1^ (amide III), indicating the formation of a β-sheet structure after gelation (Li et al., 2020). The characteristic peaks of quercetin in the range of 1,660–1,200 cm^-1^ mostly overlapped with the amide peak of SF (Song et al., 2018), but the mixture of the two caused the amide III peak (1,236 cm^-1^) of the latter to shift (Figure 1H). Based on previous studies, we speculate that hydrogen bonding between quercetin and SF leads to the shift of the characteristic peaks (Yao et al., 2020).

### 3.4 *In vitro* drug release

MMPs belong to the zinc-calcium-dependent endopeptidase family (Cabral-Pacheco et al., 2020). Under physiological conditions, the activity of MMPs in the skin is precisely regulated by their specific tissue inhibitors. However, studies on inflammatory skin lesions in patients with psoriasis have shown that the transcription and protein expression of MMP-9 are significantly increased (Simonetti et al., 2006). Thus, the project team explored the impact of MMP-9 on the drug release behavior of QSF hydrogels. In Supplementary Table S2, when the concentration of MMP-9 changed from 0 nM to 20 nM, the release rate of quercetin increased from 42.24% ± 3.99% to 63.27% ± 10.23%. To elucidate the release mechanism, fitting analyses were performed using Zero-order, First-order, Higuchi, and Korsmeyer-Peppas kinetic models (Figure 1I; Supplementary Table S3). The results indicated that although the hydrogel release behaviors with or without the addition of 10 nM MMP-9 were highly consistent with the First-order kinetic model, the K value of the 10 nM MMP-9 group was 58.40, which was significantly higher than that of the control group (41.11). This difference is mainly attributed to the enzymatic hydrolysis of fibroin by MMPs (Brown et al., 2015). The short peptide fragments generated by enzymatic hydrolysis reduced the diffusion barrier, thereby accelerating the release of quercetin. Hence, the property of QSF hydrogel to rapidly release quercetin under the trigger of exogenous MMP-9 shows good potential in the treatment application of psoriasis.

### 3.5 Potential pharmacological mechanisms of quercetin against psoriasis

Combining bioinformatics analysis and network pharmacology methods, this study investigated the mechanism and potential targets of quercetin in treating psoriasis. The 426 samples obtained from the GSE30999, GSE13355, GSE14905, GSE41662, GSE34248, and GSE50790 datasets were divided into two groups: normal and disease groups. A total of 1,301 differentially expressed molecules were identified, including 735 upregulated and 566 downregulated molecules. Differentially expressed genes were visualized in volcano plots and heat maps (Figures 2A,B). Using a Venn diagram, we screened for the common targets of quercetin and psoriasis, and initially identified 69 overlapping genes (Figure 2C). In the protein interaction network shown in Figure 2D, MMP1 and IL-1β exhibited the highest node degrees with relatively significant differential expression, indicating they are core hub targets in this network. According to GO enrichment and KEGG pathway analysis results, quercetin’s target molecules were primarily enriched in multiple psoriasis-related pathways, including the IL-17 signaling pathway, chemokine signaling pathway, TNF signaling pathway, and Toll-like receptor signaling pathway (Figures 2E–G). To further verify the regulatory effects of quercetin and its preparations on related signaling pathways, we conducted systematic *in vivo* and *in vitro* experimental studies.

**FIGURE 2:**
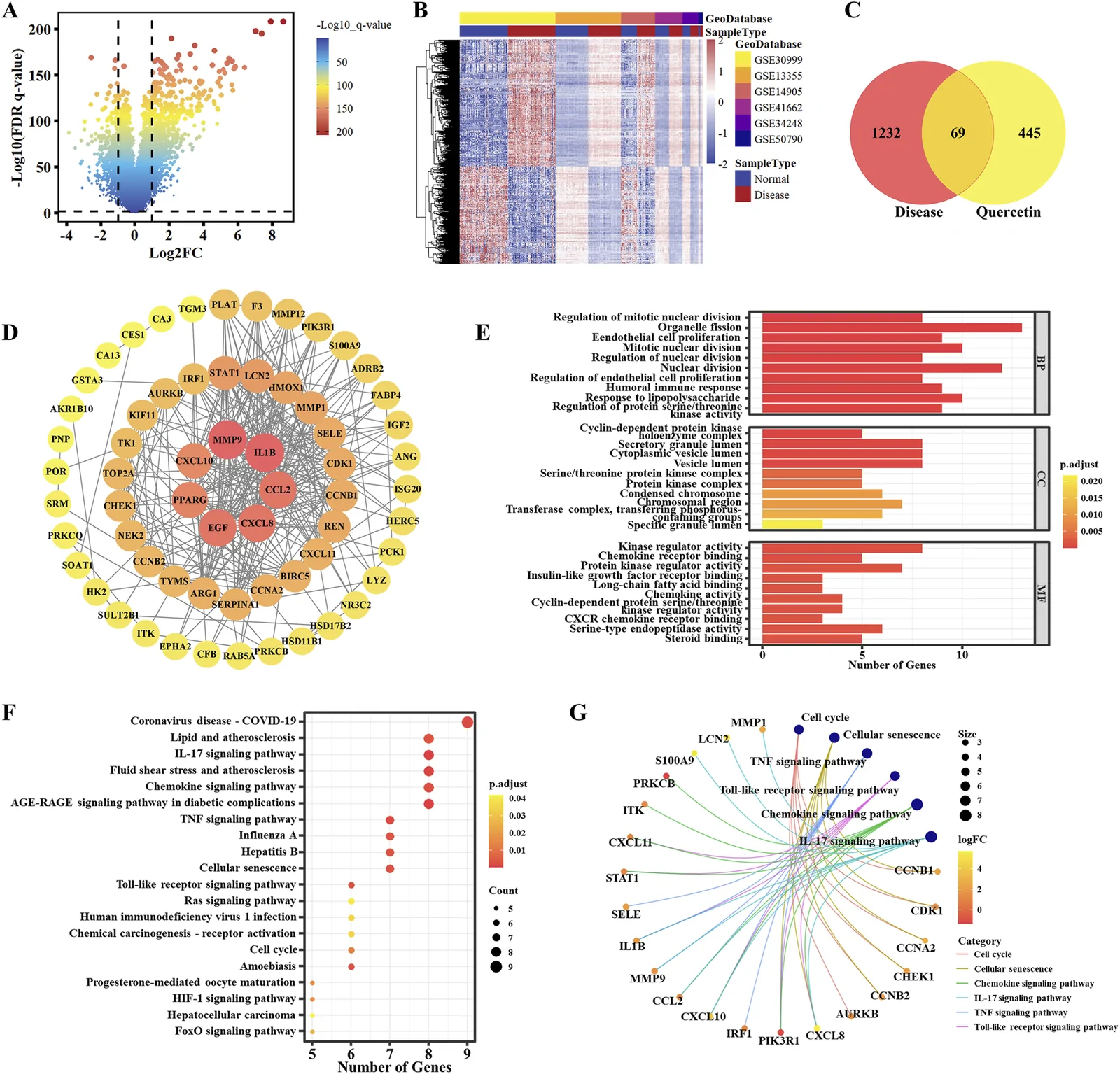
Bioinformatics analysis combined with network pharmacology analysis to identify the target of quercetin in the treatment of psoriasis. Volcano plots **(A)** and Heatmap **(B)** of DEGs between psoriasis skin tissue and normal skin tissue. **(C)** Venn diagram of the DEGs and the therapeutic target of quercetin. **(D)** PPI network diagram of psoriasis-quercetin intersection genes: the depth of color represents the level of differential expression. GO enrichment analysis **(E)** and KEGG enrichment analysis **(F)** of psoriasis-quercetin intersection genes. **(G)** Psoriasis-quercetin intersection genes and pathway association network diagram.

### 3.6 Cell proliferation and anti-inflammatory activities

Studies have demonstrated that using 100 μM imiquimod (IMQ) to induce HaCaT cells to secrete multiple pro-inflammatory factors can establish *in vitro* cell models for inflammatory skin diseases such as psoriasis (Varma et al., 2017; Váradi et al., 2024). Consequently, the CCK8 method was first used to evaluate the effects of quercetin and QSF on the viability of normal HaCaT cells and IMQ-induced psoriasis-like cells. At a concentration of 0–400 μM, after 24 h of treatment with quercetin or QSF, the viability of HaCaT cells and psoriasis-like cells remained above 80%, indicating no significant inhibition of cell growth in this concentration range (Figures 3A,B). In light of the cytotoxicity results, this work selected a drug concentration of 50 μM for subsequent anti-inflammatory activity evaluation.

**FIGURE 3:**
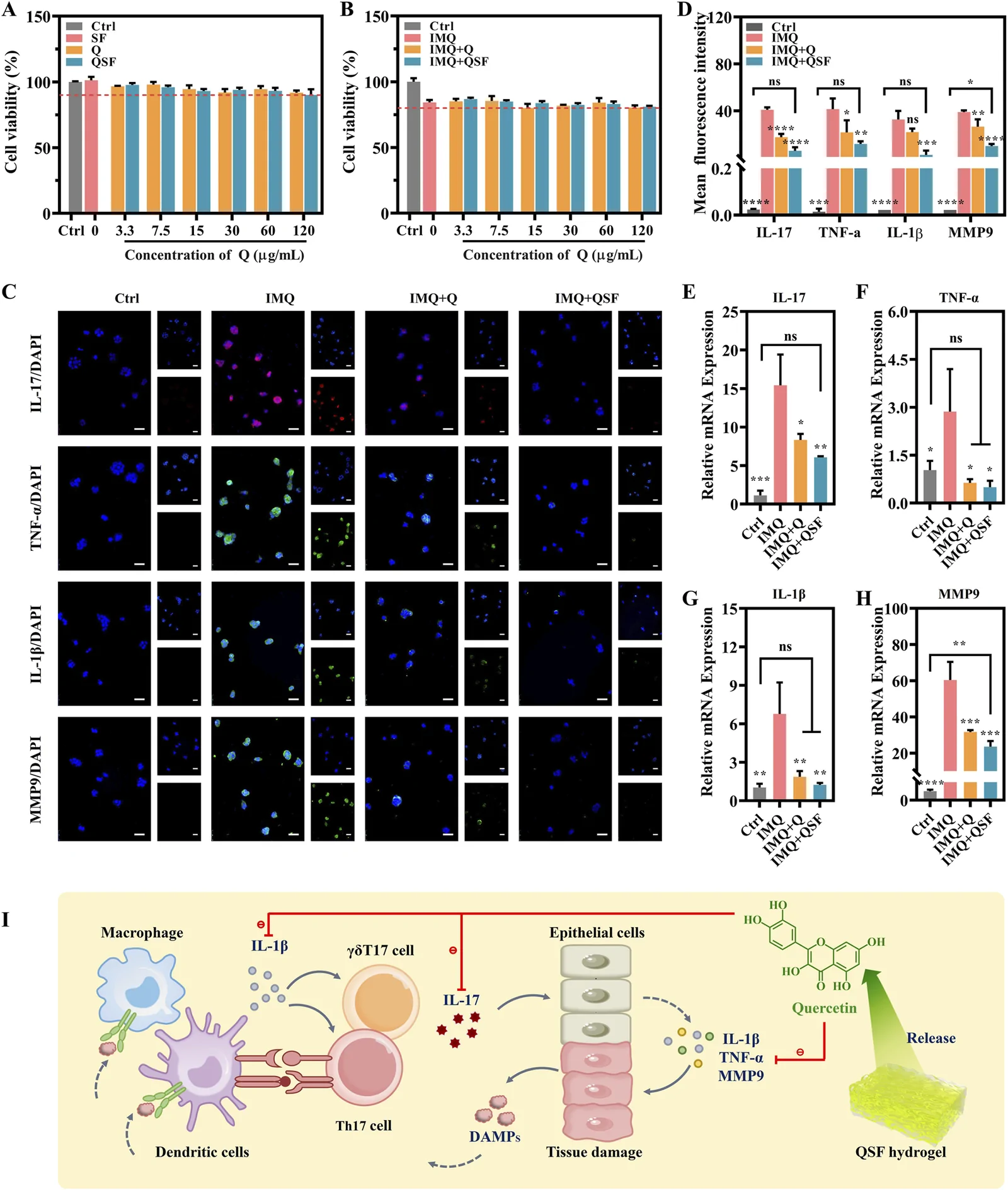
Anti-inflammatory effects of hydrogels on HaCaT cells. **(A)** Cell viability after incubation with different concentrations of quercetin (Q) or QSF was determined by the CCK-8 method. **(B)** The viability of IMQ-induced HaCaT cells after 24-h treatment with various concentrations of Q or QSF. **(C)** Representative images of IL-17/TNF-α/IL-1β/MMP-9 protein expression shown by immunofluorescence (scale = 50 μm). **(D)** The mean fluorescence intensity of IL-17/TNF-α/IL-1β/MMP-9 protein in each group was determined by ImageJ. The mRNA expression of IL-17 **(E)**, TNF-α **(F)**, IL-1β **(G)** and MMP-9 **(H)** in HaCaT cells was investigated by qRT-PCR. GAPDH was used as the endogenous control. **(I)** Schematic diagram illustrating the anti-psoriatic mechanism of the QSF hydrogel. (ns = no significance, *p < 0.05, **p < 0.01, ***p < 0.001, ****p < 0.0001 compared with the IMQ group).

In the progression of autoimmune diseases, inflammatory damage-associated molecular patterns (DAMPs) activate innate immune cells such as macrophages and dendritic cells by binding to pattern recognition receptors. This enables these cells to present exogenous peptide antigens to T cells and secrete cytokines that participate in T cell polarization (Mills, 2023). For instance, IL-1β stimulates the secretion of IL-17 by autoantigen-specific Th17 cells and γδT17 cells. IL-17 and TNF-α then synergistically act on epithelial cells (such as keratinocytes in psoriatic lesions), inducing the release of pro-inflammatory factors. Simultaneously, IL-17 additionally stimulates the production of MMPs, thereby mediating tissue damage and exacerbating inflammatory responses (Coccia et al., 2012; Alves-Filho et al., 2021). Combining literature reports with the potential targets of quercetin therapy for psoriasis predicted in Section 3.5, our research team conducted experimental validation of highly correlated genes and pathways. Immunofluorescence results indicated that IMQ treatment markedly increased the protein expression of IL-17, TNF-α, IL-1β, and MMP-9 in HaCaT cells. The mean fluorescence intensity (MFI) of representative IL-17 and MMP-9 was greatly elevated to 40.81 ± 2.02 and 38.92 ± 1.34 after IMQ induction. Upon QSF intervention, their MFI levels were prominently downregulated to 5.79 ± 3.02 and 9.89 ± 1.71 (Figures 3C,D). Similar declining trends were also observed for TNF-α and IL-1β following QSF treatment. Of note, IL-17, TNF-α and IL-1β expression in the QSF group showed no significant difference compared with the Ctrl group. In contrast, MMP-9 level in QSF-treated cells was markedly lower than that in the IMQ model group but still remained significantly higher than the control (Figures 3C,D). Consistent with the immunofluorescence findings, qPCR analysis further confirmed the transcriptional changes of these inflammatory factors. IMQ strongly induced the mRNA expression of MMP-9 up to 60.43 ± 10.00, while QSF intervention remarkably reduced its level to 23.57 ± 3.10. Likewise, QSF also effectively reversed the IMQ-induced upregulation of IL-17, TNF-α and IL-1β mRNA (Figures 3E,F), with significant differences between the IMQ and QSF groups (P < 0.05 or P < 0.01). The above results indicate that QSF can exert anti-inflammatory effects by inhibiting the IL-1β/IL-17/TNF-α axis and the activity of MMP-9 (Figure 3I).

Although cellular data demonstrated that QSF could regulate inflammatory signaling pathways and downregulate the expression of pro-inflammatory cytokines, the experimental limitations inherent to quercetin as a classic pan-assay interference compounds (PAINS) must be objectively acknowledged (Magalhes et al., 2021). Its polyhydroxy aromatic scaffold readily forms noncovalent interactions with diverse proteins, and authentic specific pharmacological effects cannot be reliably distinguished from false-positive signals based solely on *in vitro* cell models (Bolz et al., 2021). Accordingly, the current *in vitro* data have certain limitations in interpretability, and *in vivo* animal model studies are necessary to further clarify the actual therapeutic potential of the QSF hydrogel.

### 3.7 QSF hydrogel improves psoriatic skin lesions

IMQ is a potent immune activator that can establish a psoriasis animal model by topical application to the dorsal skin of mice (Park et al., 2023). To investigate the therapeutic efficacy of QSF hydrogel on psoriasis, the experimental procedures were performed as shown in Figure 4A. In brief, C57BL/6 mice were divided into four groups: The Ctrl group served as the reference, while the other three groups received 62.5 mg of IMQ applied to the dorsal skin of each mouse for 7 consecutive days. Mice in the IMQ group were not given drug treatment. Mice in the IMQ+Q group and the IMQ+QSF group were respectively given quercetin or QSF hydrogel at a dose of 120 mg/kg. Figure 4B visually illustrates the dynamic changes in the appearance of the dorsal skin across the intervention period in each group. During the disease course, the IMQ group exhibited typical psoriasis-like lesions such as prominent wrinkles, erythema, and desquamation as early as day 4 after modeling. In contrast, the experimental groups treated with quercetin or QSF hydrogel showed relatively better dorsal skin conditions, with significantly milder erythema and desquamation compared to the IMQ group. The Psoriasis Area and Severity Index (PASI) scores revealed similar overall trends in the progression of skin thickening, desquamation, and erythema among the three groups of IMQ model mice, but symptom progression was significantly restricted in drug-treated groups. Specifically, the IMQ group reached a peak total PASI score of ∼10.3 on day 5, whereas the IMQ+Q (∼7.8) and IMQ+QSF (∼6.6) groups showed significantly attenuated disease severity throughout the intervention period (Figures 4C–F). Notably, the differences in PASI sub-item scores on day 7 indicated that QSF hydrogel had comparable effects to quercetin in repairing skin erythema, while demonstrating superior efficacy in improving wrinkles and inhibiting desquamation (Figure 4G). This phenomenon suggests that silk protein hydrogel can prolong the local retention time of drugs on the skin, thereby enhancing the reparative efficacy of quercetin on psoriasis-like lesions. The weight changes of mice during the study period are shown in Figure 4H. During the first 3 days, both the model group and the treatment group exhibited weight loss. From day 4 to day 6, the weight of the model group increased rapidly, while that of the treatment group increased slowly. We hypothesize that the initial skin erythema, swelling, and pruritus induced by IMQ may reduce the mice’s appetite. As the model group mice gradually adapted to these symptoms, their appetite recovered, leading to rapid weight gain. In the treatment group, the sustained anti-inflammatory effects of the drug promoted skin repair, a process that consumed additional energy, resulting in a relatively moderate rebound in body weight.

**FIGURE 4:**
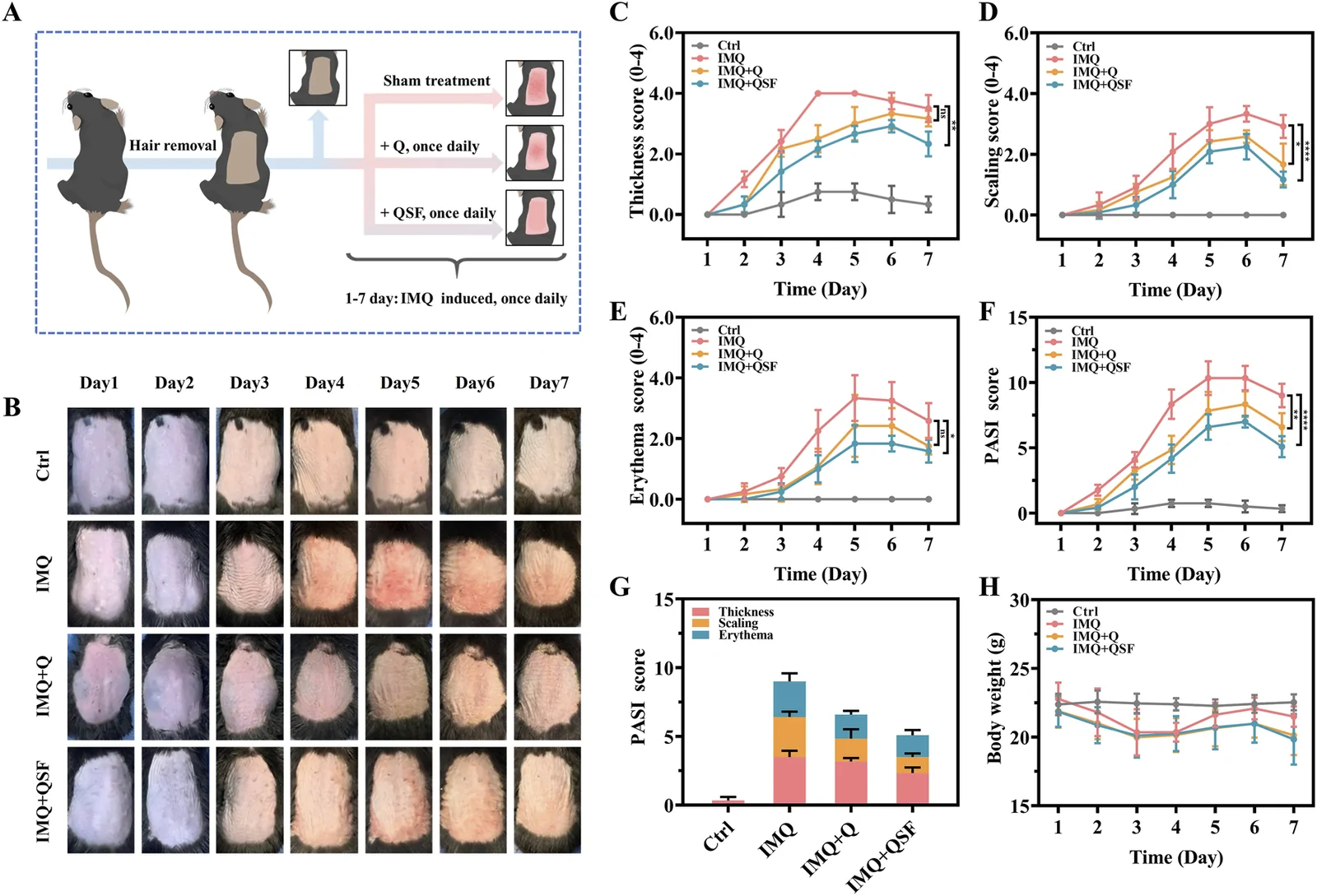
Efficacy evaluation of QSF hydrogel in IMQ-induced psoriasis animal models. **(A)** Experimental protocol for anti-psoriasis treatment. **(B)** Representative images of the backs of mice in each treated group. Disease severity was quantified by the PASI, which assessed three parameters including thickness **(C)**, scaling **(D)** and erythema **(E)**. The total PASI score **(F)** is the sum of these subscores. **(G)** The PASI scores of each group on the 7th day. **(H)** The weight changes of various mice. (ns = no significance, *p < 0.05, **p < 0.01, ***p < 0.001, ****p < 0.0001).

### 3.8 QSF alters the inflammatory milieu in skin tissues

Histopathological staining is a crucial method for evaluating pathological changes in diseases. H&E staining revealed significant pathological alterations in the skin of mice induced by IMQ, including hyperkeratosis, Munro microabscesses, acanthosis, and mononuclear cell infiltration. Compared to the IMQ group, the progressive thickening of the epidermis and dermis in the skin lesions was significantly suppressed after intervention with quercetin solution or QSF hydrogel, resulting in skin histopathological morphology more closely resembling that of normal mice (Figures 5A,B). Further analysis of H&E Baker scoring indicated that QSF hydrogel was more effective in ameliorating abnormal keratinization, with its efficacy likely closely related to the formulation characteristics of the hydrogel (Figures 5C,D). On one hand, the three-dimensional network structure of silk protein hydrogel exhibits excellent water retention capacity, alleviating symptoms such as skin dryness, hyperkeratosis, and desquamation (Olteanu et al., 2024; Kumari et al., 2025); on the other hand, the adhesive properties of the hydrogel enable long-term retention of quercetin in the lesion sites, prolonging its regulatory effects on keratinocyte proliferation and differentiation (Zaborowski et al., 2024; Yang et al., 2025).

**FIGURE 5:**
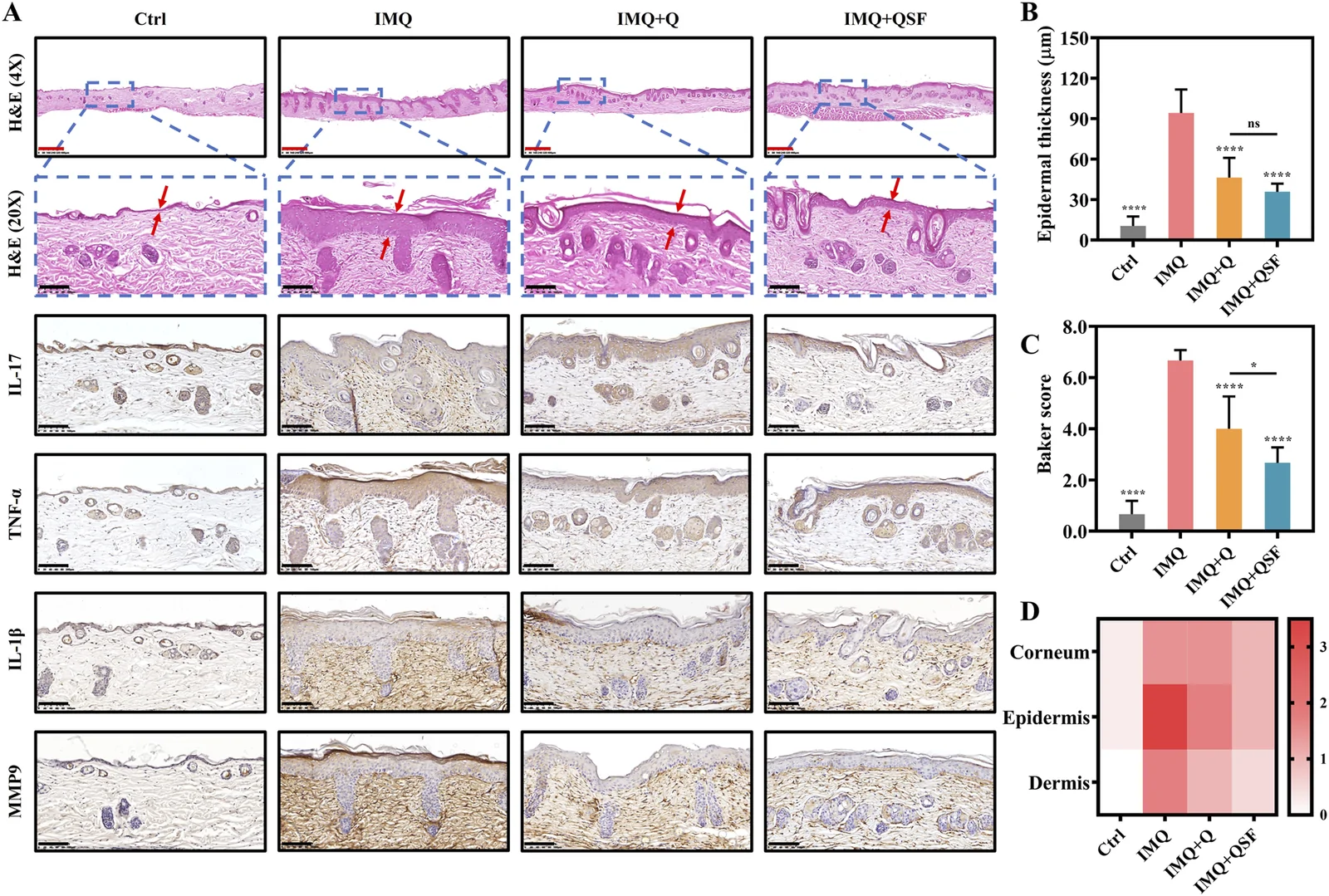
Pathological staining analysis of skin. **(A)** H&E staining and immunohistochemical staining for IL-17, TNF-α, IL-1β, and MMP-9 were performed on skin samples from the different groups (scale = 100 μm). **(B)** Epithelial thickness of skin tissues following different treatments at day 7. **(C)** The total baker scoring for evaluating the skin pathology of each group. **(D)** Heatmap of baker scores for the corneum, epidermis and dermis. (ns = no significance, *p < 0.05, **p < 0.01, ***p < 0.001, ****p < 0.0001 compared with the IMQ group).

Inflammatory factors and MMP9 play crucial roles in the pathogenesis and progression of psoriasis (Farahani et al., 2024). The results of skin immunohistochemical staining directly reflect the influence of different treatment methods on their expression levels (Figures 5A, 6A–D). Compared with the ctrl group, the positive expression levels of IL-17, TNF-α, IL-1β, and MMP9 in the skin of the IMQ group were strikingly increased. After treatment with QSF hydrogel, the positive expression areas of IL-17, IL-1β, and MMP9 in the lesion tissues were reduced to 1/3 of those in the model group, while the positive expression area of TNF-α was reduced to 1/6 of that in the model group. Additionally, the inhibitory effects of QSF hydrogel on IL-17 and TNF-α were notably superior to those of quercetin solution alone, with statistically significant differences. The results of qPCR detection of skin tissue (Figures 5E–H) showed that IMQ application could markedly induce the mRNA expression of inflammatory factors and MMP9 in the skin of mice. After treatment with the drug hydrogel, the mRNA expression levels of these factors did not differ significantly from those in the Ctrl group. In addition to immunohistochemistry and qPCR detection, ELISA kit assay results further confirmed that quercetin hydrogel effectively reduced the serum levels of IL-17 and TNF-α in mice (Figures 5I,J). Therefore, QSF hydrogel exhibits significant anti-inflammatory activity, effectively reversing the abnormal expression of inflammatory factors induced by IMQ. Its dual regulatory effects on local skin and systemic serum inflammatory factors constitute one of the core mechanisms for improving psoriasis-like lesions.

**FIGURE 6:**
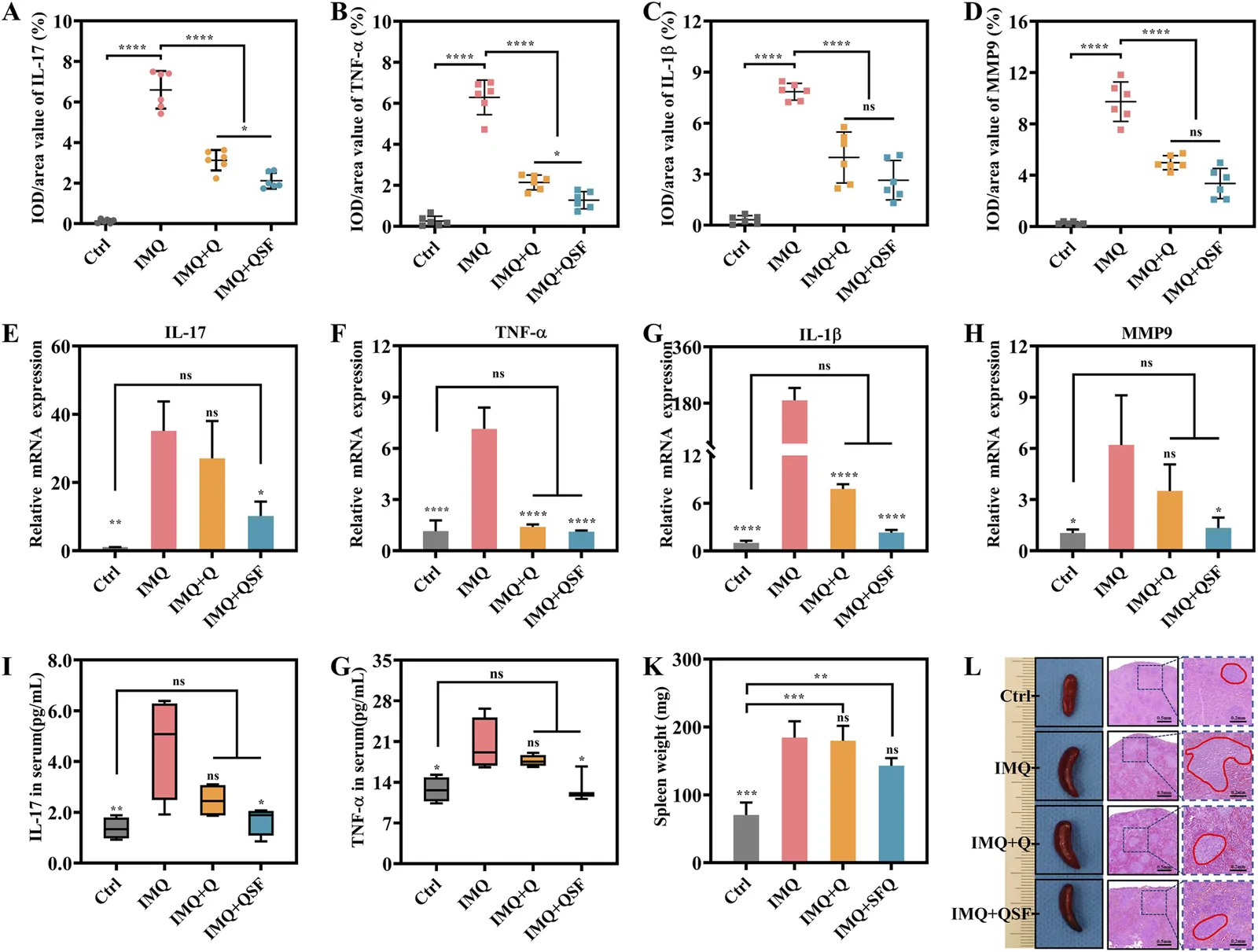
Detection of inflammatory mediators and evaluation of spleen status in mice. The immunohistochemical staining of IL-17 **(A)**, TNF-α **(B)**, IL-1β **(C)** and MMP-9 **(D)** was quantitatively analyzed by Image **(J)**. The mRNA expression of IL-17 **(E)**, TNF-α **(F)**, IL-1β **(G)** and MMP-9 **(H)** in skin tissue was investigated by qRT-PCR. The levels of IL-17 **(I)** and TNF-α **(J)** in the serum of mice in different treatment groups. **(K)** The weight of the spleen in each group of mice. **(L)** Representative appearance photos and H&E staining diagrams of the spleen. (ns = no significance, *p < 0.05, **p < 0.01, ***p < 0.001, ****p < 0.0001 compared with the IMQ group).

### 3.9 QSF-mediated immune regulation in the spleen of psoriasis mice

The application of IMQ on the skin induces systemic immune responses and splenomegaly. Therefore, on day 7 of model establishment, spleens were collected from all groups to observe and record their morphology and weight (Figure 5K). Compared with the Ctrl group, the splenic weight in the IMQ-treated group increased by 2.6-fold. Although both quercetin and its gel formulation could partially alleviate splenomegaly in the model mice, the inhibitory effect was not statistically robust compared to the IMQ group. The results of H&E staining of the spleen are shown in Figure 5L, demonstrating that the white pulp area in the IMQ group was significantly enlarged compared to the normal group (red path), whereas the white pulp area in the IMQ+QSF group showed no significant difference from the normal group. The splenic parenchyma consists of white pulp and red pulp, with the white pulp containing a higher number of lymphocytes. During immune responses, a large number of macrophages and dendritic cells are activated, leading to an enlargement of the white pulp area. These results indicate that IMQ can induce immune-associated hyperplasia of the splenic white pulp, while QSF hydrogel effectively reverses this pathological change, exerting an immunomodulatory effect.

## 4 Conclusion

In summary, this study innovatively prepared MMP-9-responsive quercetin/silk protein (QSF) hydrogel and verified its application potential in the treatment of psoriasis. This intelligent hydrogel demonstrated dual therapeutic efficacy in psoriatic lesion. Firstly, its high-water content directly acts on damaged skin, effectively alleviating superficial symptoms such as dryness and desquamation, and facilitating preliminary repair of the skin barrier. Secondly, it precisely adapts to the pathological microenvironment of psoriatic lesions with elevated MMP-9 levels, resulting in increased quercetin release at lesion sites to maximize pharmacological activity and enhance treatment efficiency. This QSF hydrogel combines biomaterials with anti-inflammatory plant metabolites, providing a new idea for the local precise treatment of psoriasis. Nevertheless, this study has certain weaknesses. These limitations include that the specificity and underlying molecular mechanisms by which MMP-9 regulates quercetin release from QSF hydrogel have not been systematically elucidated, and intervention experiments such as MMP-9 inhibitor treatment are lacking to verify the direct regulatory relationship between them. Despite these shortcomings, it preliminarily confirms a significant correlation between MMP-9 concentration and quercetin release efficiency, and validates the *in vivo* efficacy of the hydrogel. This work lays an experimental foundation for subsequent comprehensive mechanistic investigation, optimization of the drug delivery system, and further clinical translational research.

## Data Availability

The original contributions presented in the study are included in the article/Supplementary Material, further inquiries can be directed to the corresponding authors.
