# Supplementary material for: A smart MMP-9-responsive hydrogel releasing quercetin for psoriasis therapy
Source: Front Pharmacol. 2026 Sep 28;17:1810299. doi: 10.3389/fphar.2026.1810299 (PMC13655101; doi:10.3389/fphar.2026.1810299)
Supplement: Supplementary file 1 [file Table1.docx]

Supplementary Material

Figure S1. Schematic diagram of in vitro drug release

Table S1 The sequence of primers used in this study

| Gene | Primer | Base sequence (5’ to 3’) |
| --- | --- | --- |
| Human-IL-17A | Forward | TCCCACGAAATCCAGGATGC |
|  | Reverse | GGATGTTCAGGTTGACCATCAC |
| Human-TNF-α | Forward | TGCACTTTGGAGTGATCGGC |
|  | Reverse | AGCTTGAGGGTTTGCTACAAC |
| Human-IL-1β | Forward | TGATGGCTTATTACAGTGGCAA |
|  | Reverse | CCTGAAGCCCTTGCTGTAGT |
| Human-MMP9 | Forward | AGACCTGGGCAGATTCCAAAC |
|  | Reverse | CGGCAAGTCTTCCGAGTAGT |
| Human-GAPDH | Forward | AAGGTGAAGGTCGGAGTCAA |
|  | Reverse | AATGAAGGGGTCATTGATGG |
| Mouse-IL-17A | Forward | CAGACTACCTCAACCGTTCCA |
|  | Reverse | ACAATCGAGGCCACGCAGGTGCAGC |
| Mouse-TNF-α | Forward | GAGAAGTTCCCAAATGGC |
|  | Reverse | ACTTGGTGGTTTGCTACG |
| Mouse-IL-1β | Forward | GAAATGCCACCTTTTGACAGTG |
|  | Reverse | TGGATGCTCTCATCAGGACAG |
| Mouse-MMP9 | Forward | GGACCCGAAGCGGACATTG |
|  | Reverse | CGTCGTCGAAATGGGCATCT |
| Mouse-GAPDH | Forward | GAAGGTCGGTGTGAACGGAT |
|  | Reverse | ACTGTGCCGTTGAATTTGCC |

Table S2 Cumulative drug release rates of hydrogels under different MMP-9 concentrations

| The concentration of MMP-9 | Quercetin release rate |
| --- | --- |
| 0 nM | 42.24 ± 3.99% |
| 5 nM | 44.56 ± 4.55% |
| 10 nM | 58.26 ± 7.64% |
| 20 nM | 63.27 ± 10.23% |

Table S3 The release kinetics of QSF hydrogel

| Release Kinetics | | MMP(-) | MMP(+) |
| --- | --- | --- | --- |
| Zero-order | Equations | y=15.02+0.48x | y=15.48+0.76x |
|  | R2 | 0.4995 | 0.7046 |
| First-order | Equations | y=41.11*(1-e-0.15x) | y=58.40*(1-e-0.09x) |
|  | R2 | 0.9783 | 0.9866 |
| Higuchi | Equations | y=4.96*x1/2+6.97 | y=7.41*x1/2+4.51 |
|  | R2 | 0.7794 | 0.9176 |
| Korsmeyer peppas | Equations | y=13.99*x0.28 | y=13.47*x0.37 |
|  | R2 | 0.8866 | 0.9511 |
